# Annual changes in rotavirus hospitalization rates before and after rotavirus vaccine implementation in the United States

**DOI:** 10.1371/journal.pone.0191429

**Published:** 2018-02-14

**Authors:** Minesh P. Shah, Rebecca M. Dahl, Umesh D. Parashar, Benjamin A. Lopman

**Affiliations:** 1 Epidemic Intelligence Service, Office of Public Health Scientific Services, Centers for Disease Control & Prevention, Atlanta, Georgia, United States of America; 2 Division of Viral Diseases, National Center for Immunizations and Respiratory Diseases, Centers for Disease Control & Prevention, Atlanta, Georgia, United States of America; 3 Maximus Federal, Atlanta, Georgia, United States of America; 4 Department of Epidemiology, Rollins School of Public Health, Emory University, Atlanta, Georgia, United States of America; University of Liverpool, UNITED KINGDOM

## Abstract

**Background:**

Hospitalizations for rotavirus and acute gastroenteritis (AGE) have declined in the US with rotavirus vaccination, though biennial peaks in incidence in children aged less than 5 years occur. This pattern may be explained by lower rotavirus vaccination coverage in US children (59% to 73% from 2010–2015), resulting in accumulation of susceptible children over two successive birth cohorts.

**Methods:**

Retrospective cohort analysis of claims data of commercially insured US children aged <5 years. Age-stratified hospitalization rates for rotavirus and for AGE from the 2002–2015 rotavirus seasons were examined. Median age and rotavirus vaccination coverage for biennial rotavirus seasons during pre-vaccine (2002–2005), early post-vaccine (2008–2011) and late post-vaccine (2012–2015) years.

**Results:**

Age-stratified hospitalization rates decreased from pre-vaccine to early post-vaccine and then to late post-vaccine years. The clearest biennial pattern in hospitalization rates is the early post-vaccine period, with higher rates in 2009 and 2011 than in 2008 and 2010. The pattern diminishes in the late post-vaccine period. For rotavirus hospitalizations, the median age and the difference in age between biennial seasons was highest during the early post-vaccine period; these differences were not observed for AGE hospitalizations. There was no significant difference in vaccination coverage between biennial seasons.

**Conclusions:**

These observations provide conflicting evidence that incomplete vaccine coverage drove the biennial pattern in rotavirus hospitalizations that has emerged with rotavirus vaccination in the US. As this pattern is diminishing with higher vaccine coverage in recent years, further increases in vaccine coverage may reach a threshold that eliminates peak seasons in hospitalizations.

## Introduction

Prior to the 2006 recommendation of rotavirus vaccination in the United States (U.S.), rotavirus was the leading cause of severe acute gastroenteritis (AGE) in children, with characteristic annual peaks in incidence during winter-spring months [1]. The burden of rotavirus has dramatically declined with vaccination, evidenced by decreased episodes of illness, hospitalizations and emergency room visits for AGE [2–5]. Along with this decline, the epidemiology of rotavirus has also changed to exhibit a biennial pattern, evidenced by higher rotavirus incidence during rotavirus seasons (January-June) during odd years compared to lower incidence in even years [4].

This biennial pattern of rotavirus incidence peaks is unique to the U.S. among high-income countries that introduced national rotavirus vaccination programs around the same time; rotavirus incidence has fallen and remained flat in Austria, Australia, Belgium and Finland [6–9]. Vaccine effectiveness [10] and circulating strain distribution [11, 12] are similar in the U.S. and these other countries, suggesting that these factors are unlikely to explain the unique post-rotavirus vaccine epidemiology in the U.S. Compared to the other high-income countries that achieved high (84–93%) rotavirus vaccine coverage soon after introduction [13], vaccine uptake grew slowly in the U.S., with less than 70% coverage prior to 2013, when coverage reached 72.6%, and has since plateaued at 71.7% in 2014 and 73.2% in 2015 [14].

We hypothesize that the biennial pattern that emerged in the U.S., but absent in similar epidemiological settings, might be due to lower vaccine coverage. A sufficient number of susceptible children are required to sustain efficient transmission and a large seasonal epidemic. Prior to rotavirus vaccination programs, this threshold was achieved every year with each birth cohort made up of entirely susceptible children. Vaccination has reduced the annual number of susceptible children [15]. Our hypothesis is that the observed biennial peaks in rotavirus activity in the immediate years following vaccine introduction were driven by the requirement of two successive birth cohorts to accumulate a sufficiently large pool of susceptible children to drive efficient rotavirus transmission. As vaccine coverage increased in more recent years, the number of susceptible children continues to decrease each year, leading to peaks of lower magnitude.

Historical precedent for this phenomenon has been observed with measles virus in England [16]. Prior to measles vaccination, seasonal peaks in measles hospitalizations were observed every 2 years. After vaccine introduction, lower and less frequent peaks were observed until vaccine coverage reached 90% in school-aged children, at which point the peaks disappeared [17].

This hypothesis has yet to be empirically supported for rotavirus, and we would expect certain patterns to be consistent with this explanation. First, we would expect an older age distribution in the peak rotavirus seasons (odd calendar years), as the additional cases in those years would occur in those susceptible children who avoided exposure in their first year of life (a low transmission year), and became ill only when exposed in their second year of life (a high transmission year). Second, we would expect that a larger proportion of the children who became ill with rotavirus were unvaccinated in the peak rotavirus seasons than the low rotavirus seasons, evidence of the necessity of a threshold of susceptible (unvaccinated) patients to drive high transmission. Further, we would not expect to see the annual variation in hospitalizations or age prior to rotavirus vaccine introduction, and for these patterns to diminish as vaccine coverage increases in the most recent years. Using an insurance claims database, we analyzed the age distribution and vaccine coverage among children hospitalized with rotavirus and AGE during rotavirus seasons from 2002–2005 (pre-vaccine), 2008–2011 (early post-vaccine), and 2012–2015 (late post-vaccine) years. 2006–2007 were excluded as transitional years.

## Methods

### Data source

Data from the 1997–2015 Truven Health Marketscan^®^ Commercial Claims and Encounters Database were analyzed [18]. The commercial database collects data from large employers, health plans and captures de-identified patient-level data from inpatient, outpatient and prescription drug administrative claims for >230 million individuals ages 0 to 64 represented from all 50 states. Medicaid recipients are not included. In 2015, 145 employers and 15 health plans contributed to Marketscan databases. As 2002 was the first year that all age groups under 60 months were represented in Marketscan, we restricted our analysis to claims filed from January 1, 2002 –June 30, 2015. Infants residing in states with universal vaccine programs could have received rotavirus vaccination without a corresponding claim, and thus were excluded from this analysis [19]. Once a state was excluded it remained excluded even if the status of the vaccine program changed. From 2007–2012, 13 states were excluded (Alaska, Idaho, Maine, Massachusetts, New Hampshire, New Mexico, North Dakota, Oregon, Rhode Island, Vermont, Washington, Wisconsin, Wyoming). After 2013, we also added states Connecticut and South Dakota to the exclusion list.

### Hospitalization rates

Children under the age of 60 months with a hospitalization from January-June specifically for rotavirus or any AGE were eligible for inclusion in the analysis. Hospitalizations were classified by the presence of a relevant code for primary discharge diagnosis or 1 of 15 other possible discharge diagnoses in the inpatient-admissions table, similar to previous analyses of similar administrative datasets [4, 19].

As hospital coding for rotavirus is specific but may lack sensitivity [20, 21], we included International Classification of Diseases, 9^th^ Revision, Clinical Modification (ICD-9-CM) codes for both rotavirus (008.61) and AGE: viral enteritis, 008.6–008.8 (including rotavirus, 008.61); bacterial enteritis, 001.0–005.9 (excluding 003.2) and 008.0–008.5; parasitic intestinal disease, 006.0–007.9 (excluding 006.3–006.6); presumed infectious diarrhea, 009.0–009.3; presumed noninfectious diarrhea, 558.9; and diarrhea not otherwise specified, 787.91.

As birth dates are not reported in the database, the earliest claim date with the ICD-9-CM codes for live born infants, V30-V39, was used to define the enrollee’s date of birth. Age at time of hospitalization was calculated as the difference in months from the date of birth to the date of hospitalization. Age was grouped into 0–1, 2–3, 4–5, 6–11, 12–17, 18–23, 24–35, 36–47, and 48–59 months. Rotavirus seasons were defined as January-June months of each year, with even rotavirus seasons referring to 2002, 2004, 2006, 2008, 2010, 2012, and 2014 and odd rotavirus seasons referring to 2003, 2005, 2007, 2009, 2011, 2013 and 2015. Using enrollment data, we calculated person-years from the date of the birth claim until the first AGE inpatient claim or until loss of insurance enrollment or end of study period (June 30, 2015). To account for the impact of age at risk, we used the Lexis expansion to stratify each infant’s contributing person-years by age groups and follow-up period (San Hong) [22]. Seasonal rates for rotavirus and AGE admission were calculated for each age group by dividing the number of admissions by the number of person-years for all enrollees in each age group. If the hospitalization event occurred after the child was censored or lost to follow-up, then the hospitalization was excluded. Poisson regression models were used to estimate rotavirus and AGE hospitalization rates and 95% confidence intervals. Rates were calculated in SAS 9.4 (SAS Institute, Cary, NC).

### Age at hospitalization

For enrollees who were hospitalized for rotavirus or any AGE, the median and interquartile age in months at the time of hospitalization were compared during pre-vaccine, early vaccine, and post-vaccine biennial rotavirus seasons using Wilcoxon two-sample tests. To visualize the temporal course of age-stratified hospitalization rates, heatmaps were created using the *matrix* package in R (R Foundation for Statistical Computing, Vienna, Austria).

### Rotavirus vaccination coverage

Evidence for receipt of rotavirus vaccination was determined by using the Current Procedural Terminology codes 90680 and 90681 for the two rotavirus vaccines licensed in the U.S., Rotateq^®^ (RV5, Merck and Co, Whitehouse Station, NJ) and Rotarix^®^ (RV1, GSK Biologicals, Rixensart, Belgium), respectively. Vaccination coverage was calculated using a numerator of the number of enrollees with one or more claims for rotavirus vaccination prior to hospitalization and a denominator of the number of children who were age-eligible for rotavirus vaccine at the time of hospitalization for rotavirus or AGE. To be age-eligible, children were required to be at least 2 months old at the time of hospitalization and to be born after June 2006. We calculated the mean and 95% CI for vaccination coverage for all children under 60 months and stratified by age group. Vaccination coverage was compared during pre-vaccine, early vaccine, and post-vaccine biennial rotavirus seasons with Mantel-Haenszel chi-square or Fisher’s exact tests, using SAS.

## Results

### Hospitalization rates

**Fig 1:**
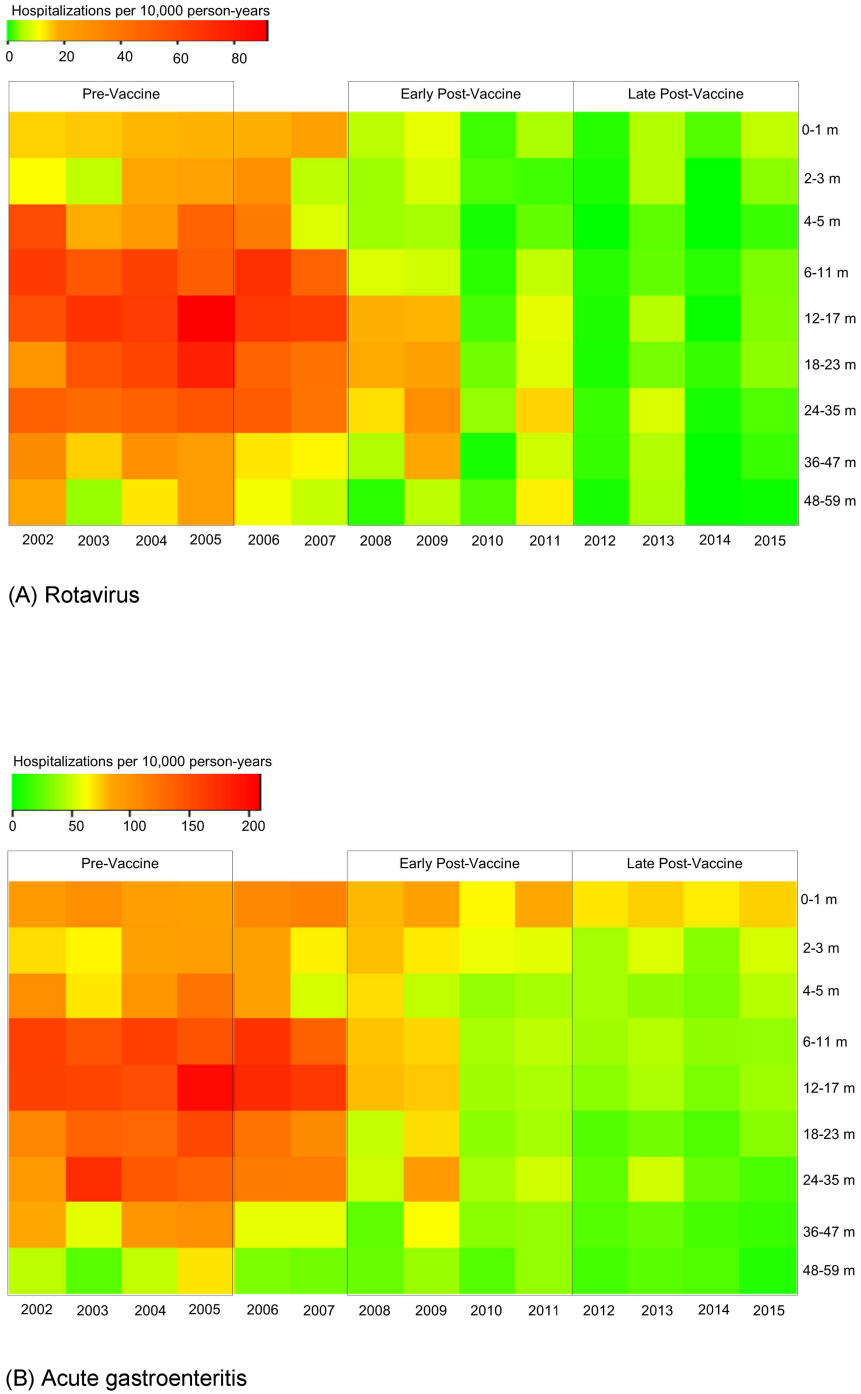
Hospitalization rates stratified by age among children <60 months. (A) Rotavirus- and (B) acute gastroenteritis- coded hospitalizations during rotavirus seasons (Jan-Jun), 2002–2015.

In a total of 2,735,860 children < 60 months of age, there were 3,172 hospitalizations coded for rotavirus and 22,712 hospitalizations coded for AGE from 2002–2015. Of all hospitalizations, 2,872 (91%) rotavirus and 13,739 (60%) AGE hospitalizations occurred during rotavirus seasons (January to June). Age-stratified hospitalization rates for rotavirus decreased from pre-vaccine to early vaccine years and further decreased during post-vaccine years (Fig 1A). In pre-vaccine years, hospitalization rates were highest in 6–23 month olds, with evidence of yearly variation but without consistent pattern. In early post-vaccine years, hospitalization rates are higher in 2009 and 2011 compared with 2008 and 2010, and especially in children >12 months old. In late post-vaccine years, the biennial pattern is not as strong; hospitalization rates are higher for older children in 2013 and 2015 compared with 2012 and 2014, but the annual variation is far more modest than in early vaccine years.

Similar to rotavirus, hospitalization rates for AGE also declined from pre-vaccine to early post-vaccine and late post-vaccine years (Fig 1B). AGE hospitalization rates during pre-vaccine years were highest in 6–35 month olds, with no consistent pattern in annual variation. In early post-vaccine years, AGE hospitalization rates were again higher in 2009 and 2011 compared to the preceding years, with higher rates particularly noticed in 2009 for 18–47 month old children. In late post-vaccine years, the biennial pattern again becomes weaker.

### Age at hospitalization

The median age at hospitalization for rotavirus hospitalizations was higher during odd seasons compared to even seasons for all three time periods evaluated (Table 1). The median age, and the absolute difference in median age in biennial seasons, was higher in early post-vaccine years (18 months in odd seasons, 14 months in even seasons) compared to pre-vaccine years (14 months in odd seasons, 12 months in even seasons). In late post-vaccine years, the difference in median age decreased to match the pre-vaccine year difference (17.5 months in odd seasons, 15 months in even seasons).

**Table 1:** Age and vaccination coverage for children < 60 months admitted for rotavirus during rotavirus seasons (Jan-Jun) before and after vaccine introduction^1^.

	Pre-Vaccine (2002–2005)			Early Post-Vaccine (2008–2011)			Late Post-Vaccine (2012–2015)		
	Odd Seasons	Even Seasons	p-value	Odd Seasons	Even Seasons	p-value	Odd Seasons	Even Seasons	p-value
**Hospitalizations, n**	667	458		426	185		165	45	
**Hospitalization rate**^2^ **(per 10,000 p-y)**	47.8	44.4	0.24	10.6	5.6	<.0001	4.0	0.96	<.0001
**95% CI**	44.3–51.5	40.6–48.7		9.7–11.7	4.9–6.5		3.5–4.7	0.72–1.29	
**Median age (mo)**	14	12	<.0001	18	14	0.0002	17.5	15	0.27
**IQR**	10–20	9–18		11–28	8–22		9–29	7–27	
**Rotavirus vaccine-eligible hospitalizations, n**	N/A			401	147		172	45	
**Vaccine coverage**				19%	15%	0.25	41%	49%	0.32
**95% CI**				15–23%	9–21%		33–48%	34–64%	

^1.^ 2006–2007 excluded as transition years

^2.^ Hospitalization rate for children 0–59 months

Abbreviation: P-y = person-years

For AGE hospitalizations, the median age was also higher in odd seasons compared to even seasons, although there was no discernible difference in this relationship during pre-vaccine, early post-vaccine, and late post-vaccine years (Table 2).

**Table 2:** Age and vaccination coverage for children < 60 months admitted for acute gastroenteritis during rotavirus seasons (Jan-Jun) before and after vaccine introduction^1^.

	Pre-Vaccine (2002–2005)			Early Post-Vaccine (2008–2011)			Late Post-Vaccine (2012–2015)		
	Odd Seasons	Even Seasons	p-value	Odd Seasons	Even Seasons	p-value	Odd Seasons	Even Seasons	p-value
**Hospitalizations, n**	1,823	1,271		2,424	1,786		1,547	1,620	
**Hospitalization rate**^2^ **(per 10,000 p-y)**	131.2	124.0		60.7	54.4		38.0	34.8	
**95% CI**	125.3–137.3	117.4–131.0		58.4–63.2	51.9–57.0		36.1–39.9	33.1–36.5	
**Median age (mo)**	13	11	<.0001	11	10	<.0001	12	10	<.0001
**IQR**	7–20	6–17		4–22	3–17		5–23	3–20	
**Rotavirus vaccine-eligible hospitalizations, n**	N/A			2,187	1,474		1,515	1,524	
**Vaccine coverage**				51%	52%	0.43	68%	71%	0.06
**95% CI**				49–53%	50–55%		66–71%	69–74%	

^1.^ 2006–2007 excluded as transition years

^2.^ Hospitalization rate for children 0–59 months

Abbreviation: P-y = person-years

**Fig 2:**
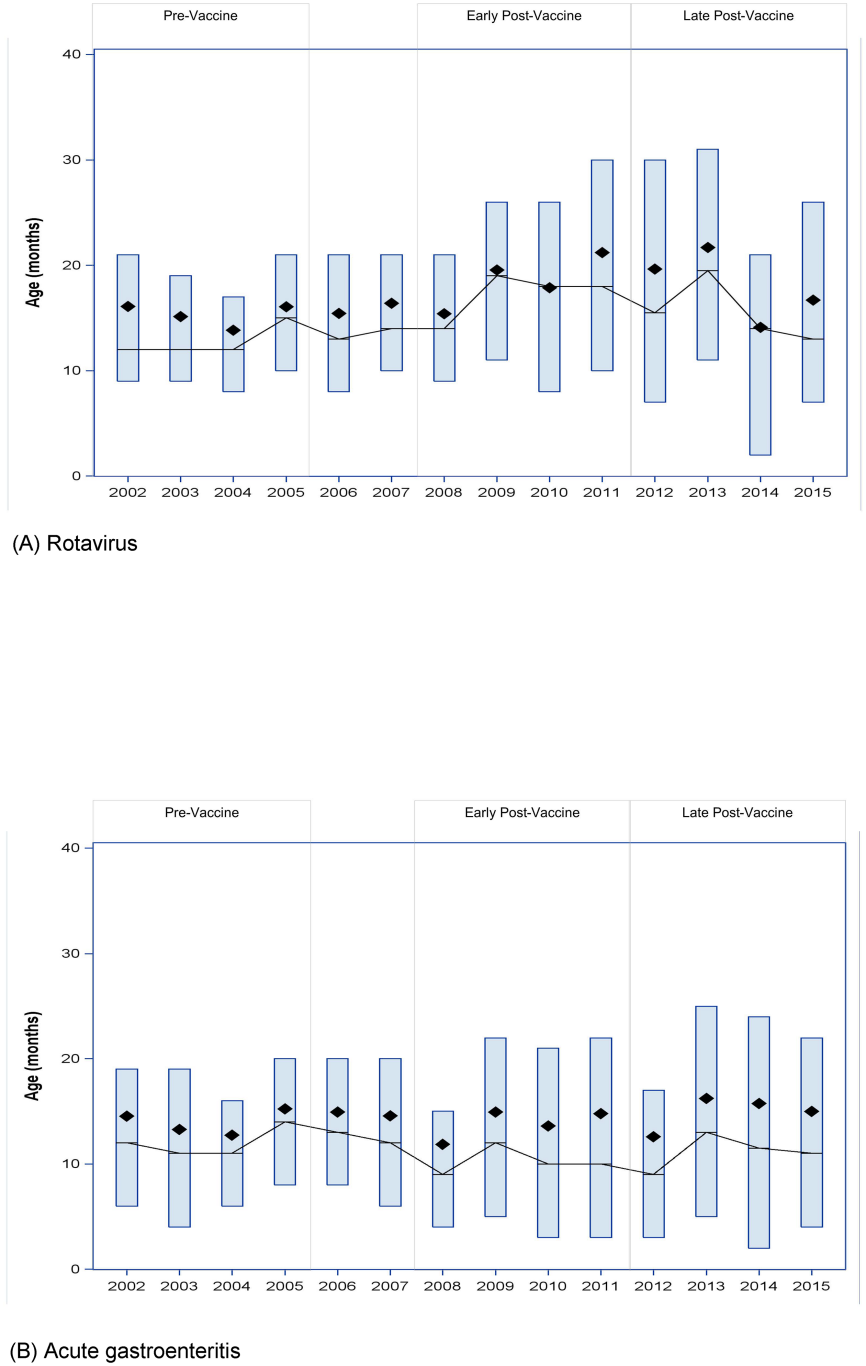
Age distribution for hospitalized children. Mean (◆), median (-), and inter-quartile range (box) of age of children <60 months hospitalized for (A) rotavirus and (B) acute gastroenteritis during rotavirus seasons (Jan-Jun), 2002–2015.

The annual variation in age at hospitalization for rotavirus (Fig 2A) and gastroenteritis (Fig 2B) did not have a consistent pattern. Age generally increased following rotavirus vaccine introduction, but was not consistently higher in odd years compared with even years.

### Rotavirus vaccination coverage

**Fig 3:**
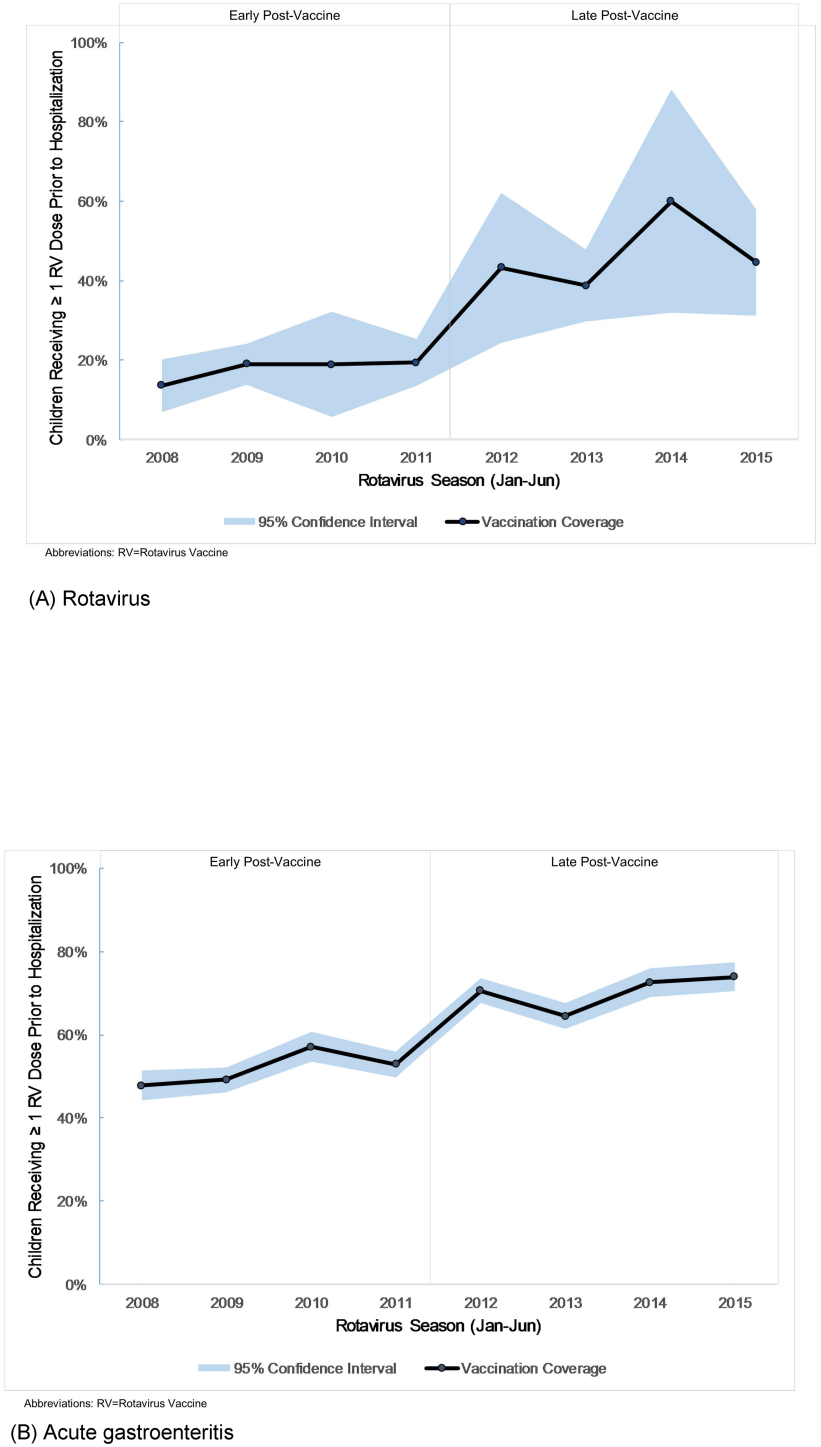
Vaccination coverage for hospitalized children. Percentage of age-eligible children < 60 months receiving at least one rotavirus vaccine dose prior to hospitalization for (A) rotavirus and (B) acute gastroenteritis during rotavirus seasons (Jan-Jun), 2008–2015.

Vaccination coverage, defined as the receipt of at least 1 rotavirus vaccination prior to hospitalization, increased in late post-vaccine years compared to early post-vaccine years, but was not different in biennial seasons for both rotavirus and gastroenteritis hospitalizations (Tables 1 and 2). Vaccination coverage generally increased over time, though there were some notable findings in the annual change in coverage. For rotavirus hospitalizations, vaccination coverage was higher in 2012 than in 2013, and in 2014 than in 2015 (Fig 3A). For gastroenteritis hospitalizations, vaccination coverage was higher in 2010 than in 2011, and 2012 than in 2013, but did not decrease in 2015 compared with 2014 (Fig 3B).

Age-stratified vaccination coverage yielded conflicting results. For rotavirus hospitalizations, vaccination coverage was not different in biennial years during both early and late post-vaccine years, with the exception of higher vaccine coverage in odd seasons for 18–23 month olds during early post-vaccine years (Table 3). For gastroenteritis admissions in early post-vaccine years, vaccination coverage was higher in even seasons for 24–35 month olds, but higher in odd seasons for 6–11 month old children (Table 4). During the late post-vaccine years, vaccination coverage was higher in even seasons for children 12–17 months and 24–47 months, but was higher in odd seasons for 2–3 month olds.

**Table 3:** Rotavirus vaccination coverage (≥ 1 rotavirus vaccination) stratified by age and rotavirus season (Jan-Jun) for children < 60 months hospitalized for rotavirus, 2008–2015. P-value comparison is for odd vs. even seasons.

	Early Post-Vaccine							Late Post-Vaccine						
Age Stratum	2008	2009	2010	2011	Even Seasons (95% CI)	Odd Seasons (95% CI)	p-value	2012	2013	2014	2015	Even Seasons (95% CI)	Odd Seasons (95% CI)	p-value
**2–3 m**	17%	14%	17%	43%	17 (0–41)%	21 (5–38)%	1.0	50%	56%	0%	40%	40 (0–100)%	50 (20–80)%	1.0
**4–5 m**	0%	38%	100%	0%	17 (0–60)%	33 (0–72)%	0.61	N/A	20%	N/A	50%	N/A	29 (0–74)%	—
**6–11 m**	23%	14%	17%	24%	22 (9–35)%	20 (10–30)%	0.81	57%	77%	75%	46%	64 (30–98)%	62 (41–82)%	1.0
**12–17 m**	13%	25%	40%	19%	16 (5–27)%	22 (13–31)%	0.39	25%	26%	100%	67%	40 (0–100)%	38 (20–55)%	1.0
**18–23 m**	4%	19%	0%	14%	3 (0–9)%	17 (9–25)%	0.04	50%	40%	57%	20%	55 (19–90)%	32 (12–52)%	0.27
**24–35 m**	0%	15%	22%	24%	20 (0–50)%	19 (11–27)%	1.0	50%	45%	50%	50%	50 (5–95)%	46 (30–63)%	1.0
**36–47 m**	N/A	N/A	0%	0%	0%	0%	—	0%	15%	N/A	33%	0%	21 (1–41)%	1.0
**48–59 m**	N/A	N/A	N/A	14%	N/A	14 (0–35)%	—	50%	22%	N/A	100%	50 (0–100)%	30 (0–65)%	1.0

Abbreviations: N/A = no admissions in the age stratum for the specified season(s)

**Table 4:** Rotavirus vaccination coverage (≥ 1 rotavirus vaccination) stratified by age and rotavirus season (Jan-Jun) for children < 60 months admitted for acute gastroenteritis, 2008–2015. P-value comparison is for odd vs. even seasons.

	Early Post-Vaccine							Late Post-Vaccine						
Age Stratum	2008	2009	2010	2011	Even Seasons (95% CI)	Odd Seasons (95% CI)	p-value	2012	2013	2014	2015	Even Seasons (95% CI)	Odd Seasons (95% CI)	p-value
**2–3 m**	39%	33%	40%	38%	40 (33–46)%	36 (30–41)%	0.33	41%	53%	36%	44%	39 (32–46)%	49 (41–57)%	0.05
**4–5 m**	63%	67%	69%	65%	66 (58–73)%	66 (59–74)%	0.89	73%	77%	83%	90%	76 (69–83)%	85 (78–92)%	0.08
**6–11 m**	54%	66%	69%	71%	60 (55–64)%	68 (64–72)%	0.005	81%	79%	85%	86%	82 (78–86)%	82 (78–86)%	0.99
**12–17 m**	48%	53%	59%	55%	52 (47–57)%	54 (49–59)%	0.60	77%	60%	86%	79%	81 (76–85)%	68 (62–73)%	0.0005
**18–23 m**	15%	37%	51%	52%	36 (28–43)%	43 (38–49)%	0.12	76%	65%	68%	73%	72 (65–79)%	68 (62–75)%	0.46
**24–35 m**	0%	33%	66%	52%	65 (55–74)%	42 (36–47)%	<.0001	68%	62%	76%	69%	72 (65–79)%	64 (59–71)%	0.11
**36–47 m**	N/A	N/A	33%	27%	33 (20–47)%	27 (17–37)%	0.44	71%	50%	75%	71%	73 (65–82)%	60 (51–69)%	0.03
**48–59 m**	N/A	N/A	N/A	28%	N/A	28 (14–41)%	—	58%	40%	67%	58%	63 (51–74)%	47 (35–59)%	0.06

Abbreviations: N/A = no admissions in the age stratum for the specified season(s)

## Discussion

Our analyses of a robust dataset of childhood hospitalizations for rotavirus and AGE provide conflicting results in evaluating the hypothesis of incomplete vaccination coverage driving the observed odd calendar year biennial peaks in rotavirus incidence in the United States since rotavirus vaccine introduction in 2006. Rotavirus and AGE hospitalization rates have decreased steadily decreased following rotavirus vaccine introduction. In early post-vaccine years (2008–2011), during which vaccine coverage slowly increased and never reached >70%, hospitalization rates were higher for older children during odd seasons. In more recent (late post-vaccine) years, annual and age-group changes in hospitalization rates are less pronounced, resulting in lower magnitude of peak seasons, and coinciding with years of higher vaccination coverage. These changes in hospitalization rates are consistent with the hypothesis of vaccine coverage driving changes in timing and size of peak rotavirus seasons.

However, annual changes in the age and vaccination coverage in children hospitalized for rotavirus and AGE are less convincing, and at times inconsistent with the changes in hospitalization rates. The age at hospitalization was higher in odd seasons compared with even seasons, even in pre-vaccine years. For rotavirus codes, the difference in age during odd seasons increased to 4 months in early post-vaccine years before returning to 2 months in late post-vaccine years, and age was higher following vaccine introduction, providing some support to the concept that peak seasons are driven by infections in older children. However, these differences were not seen in AGE hospitalizations.

Furthermore, overall vaccine coverage in hospitalized children was not higher in even seasons compared to odd seasons. Lower vaccine coverage in older (>12 month old) children during odd seasons was expected, especially in the early post-vaccine years. However, there were no differences in the vaccine coverage for any age group in any time period for rotavirus hospitalizations. For AGE hospitalizations, the late post-vaccine years had more consistent lower vaccination coverage in odd seasons than early post-vaccine years. This is an unexpected finding as the difference in hospitalization rates during biennial seasons is less pronounced in late post-vaccine years than in early post-vaccine years.

Taken together, these results provide mixed and conflicting evidence that higher hospitalization rates seen in odd years is driven by rotavirus infections in older, unvaccinated children who are being exposed to rotavirus at an older age than they would have been prior to vaccine introduction. While vaccination coverage has a role in the changing pattern of rotavirus hospitalizations, other factors, such as differential susceptibility of older children to the dominant circulating rotavirus genotypes since vaccine introduction [23], should be considered, although the genotype distribution has not followed a similar biennial pattern.

Our study has some limitations. First, uninsured and Medicaid populations are not represented in MarketScan data, which may affect generalizability of our findings. Medicaid recipients may have lower childhood vaccination coverage than commercial insurance [14], and thus could have stronger biennial patterns than observed in this analysis. However, this being a time series analysis, we are most concerned with time varying biases. Second, changes in hospitalization rates could be driven by changes in admitting patterns or insurance coding rather than true illness. It is reassuring that we found a similar biennial pattern for hospitalization rates, age at hospitalization and vaccination coverage as has been seen for laboratory rotavirus detection and hospitalizations in other studies using different data sources [2–4]. Third, herd immunity and indirect protection of unvaccinated children complicate proving direct causal association between vaccination coverage and disease patterns [24]. Fourth, the large declines in rotavirus and gastroenteritis hospitalizations following vaccine introductions have led to a small number of observations when stratified by age and year. Thus, vaccination coverage estimates in Tables 3 and 4 have large confidence intervals, especially in recent years. The low number of observations further precludes disaggregation by state or region, vaccine type, or incomplete vaccination series.

In conclusion, these results show that the biennial peak in U.S. rotavirus and AGE hospitalizations since vaccine introduction could be driven in part by incomplete vaccination, and therefore the build-up of susceptible children, in consecutive birth cohorts. However, incomplete vaccination is not a sufficient and complete explanation, and other factors should be investigated. The biennial pattern that emerged in the U.S. following vaccine introduction may be beginning to diminish in magnitude during peak seasons. It is possible that this pattern will continue to change with increased rotavirus vaccination coverage, and perhaps reach a threshold that prevents peaks altogether.

## Supporting information

S1 Table Data file. Data file for Marketscan rotavirus and acute gastroenteritis hospitalization codes, 2002–2015.
(ZIP)
